# Supplementary material for: Netcombin: An algorithm for constructing optimal phylogenetic network from rooted triplets
Source: PLoS One. 2020 Sep 18;15(9):e0227842. doi: 10.1371/journal.pone.0227842 (PMC7500971; doi:10.1371/journal.pone.0227842)
Supplement: S4 File — (ZIP) [file pone.0227842.s004.zip › SequenceData-Triplet/sequenceData-10-20-30-0-Triplet/length 200, numseq 40, rate 10^-7/8 seq.pdf]

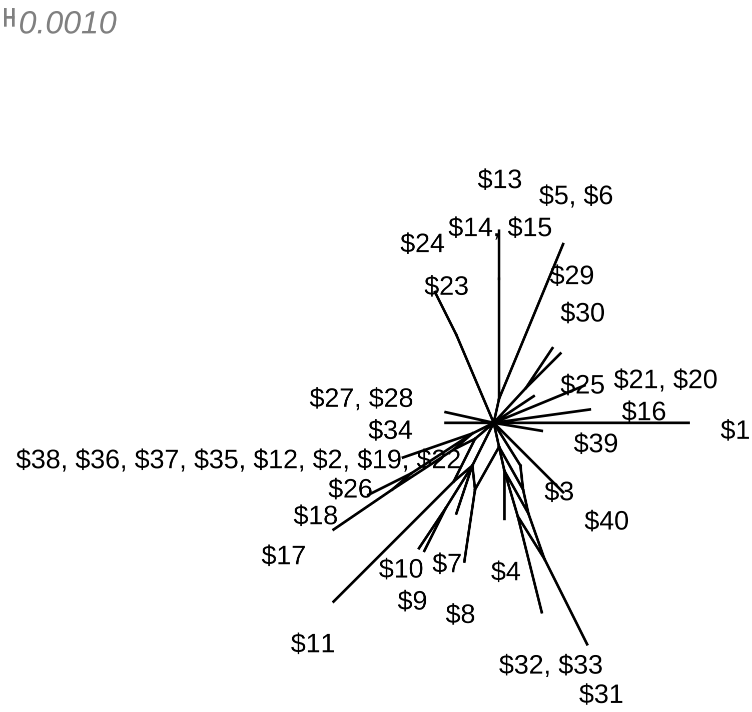

0.0010
$13
$5, $6
$14, $15
$24
$23
$29
$30
$25
$21, $20
$16
$1
$27, $28
$34
$39
$38, $36, $37, $35, $12, $2, $19, $22
$26
$3
$18
$40
$17
$10
$7
$4
$9
$8
$11
$32, $33
$31
